# Phenomics Study Reveals Predictable Converging Molecular Signatures in CTE, Alzheimer's and Models of Mild TBI

**DOI:** 10.1002/alz70855_099078

**Published:** 2025-12-23

**Authors:** Zezong Gu, Marcus Jackson, Tamanna Mony, Wenyan Yu, Thi Thao Nguyen, Pei Liu, Shanyan Chen, Runting Li, Gregory M Cole, Sally A. Frautschy, Jiankun Cui

**Affiliations:** ^1^ University of Missouri, Columbia, MO, USA; ^2^ HARRY S. TRUMAN MEMORIAL VA HOSPITAL, Columbia, MO, USA; ^3^ University of Missouri School of Medicine, Columbia, MO, USA; ^4^ University Of Missouri Columbia, Columbia, MO, USA; ^5^ Veterans Greater Los Angeles Healthcare System, Los Angeles, CA, USA; ^6^ Mary S. Easton Center for Alzheimer's Disease Research at UCLA, Los Angeles, CA, USA; ^7^ University of California Los Angeles, Los Angeles, CA, USA; ^8^ Harry S. Truman Memorial Veterans’ Hospital, Columbia, MO, USA

## Abstract

**Background:**

Traumatic brain injury (TBI) accelerates risk for multiple Alzheimer's Disease and Related Dementias (ADRDs) featuring Tau protein hyperphosphorylation and tauopathy. Mild TBI (mTBI) causes a temporary disruption of brain function typically caused by blast, blow, or jolt to the head. While many individuals recover fully within a few weeks or months, some may experience long‐term cognitive deficits, but how TBI progresses to ADRDs including tauopathies remains complex and elusive. The present study identified the combinations of risk genes and timing of mTBI induced by open‐field blasts (OFB) that alter candidate fluid and imaging biomarkers involved, so that making early intervention possible to prevent or delay the progression of ADRD.

**Method:**

human wild‐type Tau/CamKII bitransgenic (rT1) and Non‐carrier/non‐carrier mice were assigned randomly into two groups: OFB‐induced repetitive mTBI and sham control and evaluated for transgene and TBI‐dependent behavioral deficits at 3 months post‐injury. The brain tissues were collected for proteomics studies using high‐resolution label‐free global and phospho‐proteomics by liquid chromatography coupled with tandem mass spectrometry, followed by A.I. informed phenomic analysis integrating behavioral‐related proteomic datasets with human databases. Immunoblotting validated selected phenomics findings.

**Result:**

We identified co‐expression subnetworks that were strongly correlated with PTSD‐like behavioral endophenotypes in humans including psychomotor agitation, fear response, and physical activity in rT1 mice following OFB‐induced repetitive mTBI. Biologically‐informed neural networks (BINN)‐enhanced eXplainable Artificial Intelligence (XAI) analysis of the differential expression (DE) profiles in human ADRD cohorts that differentiate symptomatic CTE and AD from asymptomatic AD and age‐matched human controls, identified overlapping networks in both mouse and human studies. Key proteins that regulate synaptic vesicle cycling, synaptic plasticity, and energy metabolism displayed DE in rT1 mice as a function of tau transgene and TBI interactions and drivers to accelerate their trajectory towards ADRD. Several of the TBI‐triggered DE proteins (YWHAG or 14‐3‐3 γ, HNRNPA2B1, and hexokinase), overlapped reported phosphoTau and Tau oligomer interactomes with proposed causal roles in metabolic deficits and neurodegeneration.

**Conclusion:**

This study unveiled ADRD predictable converging molecular signatures that appear to drive the Tau‐TBI interaction conferring both chronic neuropsychiatric impairments and neurodegenerative disease progression, shedding light on the complex etiology of ADRD.

